# Everybody's Page

**Published:** 1888-10-27

**Authors:** 


### Everybody's Page.

A remarkable condition, known as night-blindness, often results from retinal exhaustion, due to protracted exposure to bright light; and many interesting cases have been placed on record.

Of all games football exposes the player to the greatest risk of injury; the more rapid movement and closer contact of the bodies of the players in this game, of necessity increases the chance of injury. The gravity of the injuries is usually greater in this than in any other game or sport.

Carbolic acid was known even in the days of Pliny, who states that it was prepared by the destructive distillation of wood, and that the liquor thus obtained was reboiled, and the heavy pitch oil collected on fleeces of wool. He talks of it as a "reddish pitch, very clammy, and much fatter than other pitch."

Play in a child means health, and makes health. Sleep in abundance is most necessary during development. A child can hardly sleep too long, especially one brought up in a city. But the minimum of sleep should be 12 hours a-day up to 4 years, 11 hours from that to 7 years, 10½ from 7 to 10, 10 hours from that to 15, and 9 up to 20 years of age.

The sound of one's own voice in the head, double hearing, or autophony, is a phenomenon by no means rare in sundry diseases of the ear. Sir Everard Home states that an eminent musicmaster once, after catching a cold, for a long time found that the pitch of one ear was half a note lower than that of the other, and that the perception of a simple sound did not occur in both ears at the same instant, but was rendered double, the second sound being the lower and weaker.

Gas spoils the air to a much greater extent than other means of illumination, not only using up more oxygen, but yielding by its combustion a considerable volume of sulphurous acid and other gases, which are extremely deleterious, even in small quantities. The effects of these gases are quickly shown in conservatories by the drooping of plants, and make themselves apparent in rooms by their action on the leather binding of books.

Cheerful company at meals is by no means an unimportant addition to healthy digestion. It is, on the contrary, one which is too frequently overlooked, to the detriment of health. It is true this may be a condition not always or readily attainable; but if "a merry heart lives long," as the poet has expressed it, it forms a justification of the aphorism that the happiness thus induced must both act and react upon the healthy digestion of food.

Special genius for art is hereditary. A very good example of this is seen in the family of Titian, which included nine painters of merit. According to Ribot, the family of Sebastian Bach is one of the most distinguished instances of mental heredity on record. During a period of nearly 200 years, this family produced a multitude of musicians of the first rank. They were all organists and church singers, or belonged to municipal orchestras.

A favourite Persian cat, on friendly terms with many households, was able to spread scarlet fever amidst quite a large community of children in Chicago last year. This animal had been petted by a child lying in bed ill with the disease, and in its subsequent visits to several neighbours' houses it carried the disease with it. Nothing is more natural than that its thick fur should harbour infective particles from the patient in large quantities, and nothing more likely than that these should be removed and wafted through the air every time its fur was stroked afterwards.

There are several chemical substances which prove injurious to the workers in particular trades. Lucifer matchmakers, for example, used not unfrequently to suffer from decay (necrosis) of the jaw-bone, the result of phosphorus poisoning. Painters, plumbers, and all persons who come in contact with white lead, are liable to suffer from chronic lead poisoning. The manufacturers of white lead not unfrequently die from lead poisoning.

Dirty houses, dirty clothing, dirty companions, lower a man both morally and physically; they lower him in his own estimation, as well as in the opinion of others. Personal cleanliness and a tidy house increase a man's self respect, companions of a like mind are calculated to stimulate to a similar course of action. The old-fashioned tub-night is a most healthful and important national institution, and along with the clean linen and better clothing worn on the Sunday, does much to maintain the self-respect of the people.

The friends of a deaf child have sometimes not the slightest notion that it is hard of hearing; and, reflecting on the faults and corruption of their own nature in youthful days, they are apt to inform the little sufferer that it is careless or provokingly obstinate, and that "None is so deaf as he that won't hear." We read in Holmes's "System of Surgery" of a boy who from early life, had been the subject of aural disease, and who actually died from abscess in the brain caused thereby, but whose father used to box his ears for what he termed "inattention."

The first sign of conscience, or a knowledge of right and wrong, was noticed by Darwin at the age of thirteen months, when his boy was made to look and feel unhappy by being accused of not giving his papa a kiss. A certain tendency to deceitfulness, and to do furtively what they have been told not to do and know to be wrong, is natural to most children, but it is developed in some to an enormously greater extent, and much sooner than in others. The children of the habitual criminal classes are said on all hands to take to deceit as a duck takes to water.

Even when a corset is not tight enough to compress internal organs, it prevents the external muscles of the body from acting as they ought, so that in time they become feeble from want of use, like the muscles of an arm which has been bound up in a splint. There are about seventeen little joints, too, in the spine, which are more or less immovably fixed by the wearing of a stiff whalebone down the back, so that it is difficult to understand what people mean who insist that loose stays can do no harm. If a garment that is fixed over those flexible joints is either tight or stiff, it must do some harm, and if it is neither one nor the other, it cannot be called a corset.

Their capacity for reproduction enables the eyes to resist apparently very serious injury. Thus, several cases are on record in which lead-workers having poured molten lead into a cavity containing a drop or two of water have caused an explosion, the water becoming suddenly converted into steam. A splash of the hot metal has been propelled into the eye, and, owing to its liquid condition, has spread over its surface as a thin plate between the eye and the eyelid, forming, in fact, a perfect mould of the front of the eye. Yet, in some cases, though it might be inferred that the destruction of the eye would be certain and speedy, it has been found that on removal of the metal the eye has remained perfectly clear, or, at most, suffered from temporary inflammation, with loss of some of the superficial cells, which have been soon reproduced.

